# Supplementary material for: GPR15LG binds CXCR4 and synergistically modulates CXCL12-induced cell signaling and migration
Source: Cell Commun Signal. 2025 May 20;23:234. doi: 10.1186/s12964-025-02231-x (PMC12093852; doi:10.1186/s12964-025-02231-x)
Supplement: Supplementary file 5 — Supplementary Material 5 [file 12964_2025_2231_MOESM5_ESM.docx]

Computer simulations of GPR15LG with the CXCR4 or the ACKR3 receptor. GPR15LG was initially placed 100 Å away from the center of mass of the receptor with three different orientations (see Supplementary Fig. S1). The videos illustrate 9 ns of cMD followed by 40 ns of GaMD equilibration and 400 ns of GaMD production (one replica in each case). Ions and water molecules are not shown for clarity. The lipid membrane is only shown in the first frame, also for simplicity.
